# Biological Activity and Molecular Structures of Bis(benzimidazole) and Trithiocyanurate Complexes

**DOI:** 10.3390/molecules200610360

**Published:** 2015-06-04

**Authors:** Pavel Kopel, Dorota Wawrzak, Vratislav Langer, Kristyna Cihalova, Dagmar Chudobova, Radek Vesely, Vojtech Adam, Rene Kizek

**Affiliations:** 1 Department of Chemistry and Biochemistry, Faculty of Agronomy, Mendel University in Brno, Zemedelska 1, Brno CZ-613 00, Czech Republic; E-Mails: kriki.cihalova@seznam.cz (K.C.); dagmar.chudobova@centrum.cz (D.C.); vojtech.adam@mendelu.cz (V.A.); kizek@sci.muni.cz (R.K.); 2 Central European Institute of Technology, Brno University of Technology, Technicka 3058/10, Brno CZ-616 00, Czech Republic; 3 Institute of Chemistry, Environmental Protection and Biotechnology, Jan Dlugosz University of Czestochowa, Armii Krajowej 13/15, Czestochowa PL-42-201, Poland; E-Mail: d.wawrzak@ajd.czest.pl; 4 Environmental Inorganic Chemistry, Department of Chemical and Biological Engineering, Chalmers University of Technology, Göteborg SE-412 96, Sweden; E-Mail: langer@chalmers.se; 5 Department of Traumatology at the Medical Faculty, Masaryk University and Trauma Hospital of Brno, Ponavka 6, Brno CZ-662 50, Czech Republic; E-Mail: ves.radek@seznam.cz

**Keywords:** coordination compounds, biological activity, trithiocyanuric acid, trimercaptotriazine, single crystal X-ray diffraction, benzimidazole

## Abstract

1-(1*H*-Benzimidazol-2-yl)-*N*-(1*H*-benzimidazol-2-ylmethyl)methanamine (**abb**) and 2-(1*H*-benzimidazol-2-ylmethylsulfanylmethyl)-1*H*-benzimidazole (**tbb**) have been prepared and characterized by elemental analysis. These bis(benzimidazoles) have been further used in combination with trithiocyanuric acid for the preparation of complexes. The crystal and molecular structures of two of them have been solved. Each nickel atom in the structure of trinuclear complex [Ni_3_(abb)_3_(H_2_O)_3_(μ-ttc)](ClO_4_)_3_·3H_2_O·EtOH (**1**), where **ttc**H_3_ = trithiocyanuric acid, is coordinated with three N atoms of **abb**, the N,S donor set of **ttc** anion and an oxygen of a water molecule. The crystal of [(tbbH_2_)(ttcH_2_)_2_(ttcH_3_)(H_2_O)] (**2**) is composed of a protonated bis(benzimidazole), two **ttc**H_2_ anions, **ttc**H_3_ and water. The structure is stabilized by a network of hydrogen bonds. These compounds were primarily synthesized for their potential antimicrobial activity and hence their possible use in the treatment of infections caused by bacteria or yeasts (fungi). The antimicrobial and antifungal activity of the prepared compounds have been evaluated on a wide spectrum of bacterial and yeast strains and clinical specimens isolated from patients with infectious wounds and the best antimicrobial properties were observed in strains after the use of ligand **abb** and complex **1**, when at least 80% growth inhibition was achieved.

## 1. Introduction

Heterocyclic compounds are very often used in the development of novel drugs with new mechanisms of action. Among heterocyclic compounds, arylaziridines [1] and benzimidazole derivatives have very important role because of their wide spectrum of biological activities. Benzimidazole and its derivatives are reported to be physiologically and pharmacologically active and some applications are found in the treatment of several diseases including epilepsy, diabetes and infertility. These compounds show a wide range of biological activities like antibacterial, antifungal, antitubercular [2], antimalarial [3], anti-inflammatory, analgesic, antiamoebic [4] antihistamine [5], antiulcerative, antioxidant [6,7], antiproliferative [8], antihypertensive [9], antiallergic [10], antitumor [11,12,13], antikinase and anti-HIV-1 properties [14]. Some benzimidazoles have also been evaluated as cholinesterase inhibitors [15,16] and drugs against parasites [17,18].

**Figure 1:**
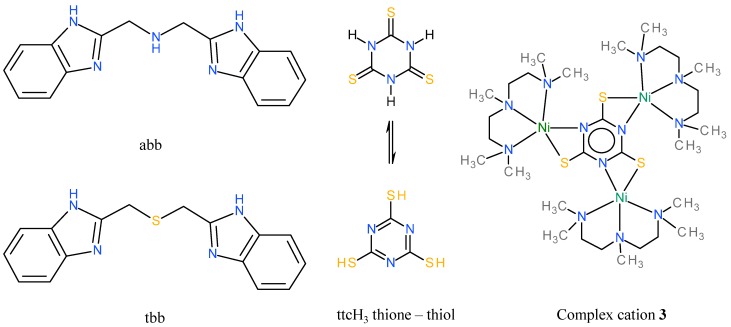
Structural formulas of the ligands used and the complex cation [Ni_3_(pmdien)_3_(μ-ttc)](ClO_4_)_3_ (**3**). **abb** = 1-(1*H*-benzimidazol-2-yl)-*N*-(1*H*-benzimidazol-2-ylmethyl)methanamine, **tbb** = 2-(1*H*-benzimidazol-2-ylmethylsulfanyl-methyl)-1*H*-benzimidazole, **ttc**H_3_ = trithiocyanuric acid in its thione and thiol forms.

Trithiocyanuric acid (**ttc**H_3_), also referred as trimercaptotriazine, has a symmetric structure with three nitrogen atoms and three sulphur atoms in a ring (see Figure 1). These donor atoms can be used for coordination to central atoms and can also form bridges among central atoms. Trithiocyanuric acid is used as remediation agent for the removal of heavy metals from industrial wastewaters [19,20]. A very important application of **ttc**Na_3_ is the removal of residual palladium from reaction mixtures, especially in drug production [21,22]. It was also applied in plating processes, in the production of composite materials containing metals and rubbers and as an anticorrosion agent [23,24].

The biological activity of trithiocyanuric compound was also evaluated as it can serve as a ligand of *Toxoplasma gondii* orotate phosphoribosyltransferase [25]. Trinuclear ruthenium complexes including [(Ru(L)_2_)_3_(μ-ttc)](ClO_4_)_3_ (L = phenylazopyridine) with proved tris-S,N chelating mode were characterized by Kar *et al.* [26,27]. The interactions of the trinuclear complexes with the circular and linear forms of *p*-Bluescript DNA show reduced ethidium bromide fluorescence on gel electrophoresis. Some very interesting polynuclear zinc complexes with macrocyclic ligands and **ttc**^3−^ were studied by Aoki *et al.* [28,29,30]. The (Zn_4_(L_4_)_3_(ttc)_4_)^12+^ complex (L = cyclen = 1,4,7,10-tetraazacyclododecane), with a trigonal prism configuration is very stable in aqueous solution at neutral pH. The complex does not dissociate into the starting building blocks in the presence of Zn^2+^ binding anions such as phosphates and double-stranded DNA. The results of the competitive binding assays with ethidium bromide and calf-thymus DNA, thermal melting experiments, and gel mobility shift assays altogether indicated that the complex induced the aggregation of double-stranded DNA. The antitumor and antimicrobial activities were evaluated for Ni(II), Fe(II) and Mn(II) trithiocyanurate complexes [31]. The antitumor activity of the complexes was assayed *in vitro* against G-361 (human malignant melanoma), HOS (human osteogenic sarcoma), K-562 (human chronic myelogenous leukaemia) and MCF-7 (human breast adenocarcinoma) tumour cell lines. The IC_50_ values of the Fe(II) and Mn(II) compounds turned out to be lower than those of cisplatin and oxaliplatin. The complexes of Fe(II), Mn(II) and Ni(II) with a combination of a Schiff base, nitrogen-donor ligand or macrocyclic ligand and trithiocyanuric acid were prepared and their anticholinesterase activity was studied [32]. It was shown that the tested compounds inhibit cholinesterases and can be considered as potential drugs for Alzheimer’s disease or as prophylactics in the case of nerve agent exposure or poisoning by pesticides.

In this paper, we present the possible application of some bis(benzimidazole) (their formulas are given in Figure 1) and trithiocyanuric complexes in the treatment of severe and difficult to treat bacterial or yeast infections. The major aim of this study was to test the biological activity of these compounds on the commercially supplied microorganisms (*Staphylococcus aureus*, *Escherichia coli* and *Saccharomyces cerevisiae*) and three bacterial strains isolated from patients from non-healing wounds infected by bacterial strains (*Streptococcus agalactiae*, *Corynebacterium striatum* and *Serratia marcescens*). For the microbiological testing, an already known and well characterized coordination compound, [Ni_3_(pmdien)_3_(μ-ttc)](ClO_4_)_3_ (**3**), where **pmdien** = *N*,*N*,*N*′,*N*′′,*N*′′-pentamethyldiethylenetriamine, was also prepared according to published method [33]. The complex **3** has a similar structure (see Figure 1) to **1** and it was of interest to compare their biological activities. The successful preparation of the ligands was unambiguously proved by single crystal X-ray analysis of two compounds.

## 2. Results and Discussion

### 2.1. Synthesis and Characterization

Bis(benzimidazole) ligands **abb** and **tbb** were prepared by well-known methods. This method is time consuming but it gave good yields, especially in the case of **tbb**. The composition of both compounds was confirmed by elemental analyses and moreover by single crystal X-ray analysis of reaction product with nickel salt and **ttc**H_3_, respectively. The trinuclear complex [Ni_3_(abb)_3_(H_2_O)_3_(μ-ttc)](ClO_4_)_3_·3H_2_O·EtOH (**1**) was prepared by the reaction of nickel perchlorate, **abb** ligand and very slow addition of **ttc**Na_3_ in an ethanol‒water mixture. Previously, we used the same strategy for the preparation of similar trinuclear complexes just like in case of [Ni_3_(pmdien)_3_(μ-ttc)](ClO_4_)_3_ (**3**). Without the presence of terdentate ligand **abb** a polymeric poorly soluble precipitate should be formed. The perchlorate salt was shown to be a good counterion in preparation of bi- or trinuclear complexes. The composition of **1** was proposed on the basis of the elemental analysis and confirmed by single crystal X-ray analysis. The formation of co-crystals between organic compounds containing N, S or O atoms is well known. For example, Ranganathan characterized channel structures of melamine with **ttc**H_3_ [34] and recently co-crystals of N-donor ligands (4,4′-bipyridine, 1,2-bis-(4-pyridyl)ethylene, 1,7-phenanthroline) and **ttc**H_3_ were reported by Nagarajan [35]. In our work, we succeeded in preparation of [(tbbH_2_)(ttcH_2_)_2_(ttcH_3_)(H_2_O)] (**2**) co-crystals by reaction of **tbb** with **ttc**H_3_ in EtOH solution. The formation of **2** was also confirmed by X-ray structural analysis. The solubility of all compounds was checked. All the compounds are soluble in methanol, ethanol, DMSO and DMF and insoluble in water. For the biological testing and cyclic voltammetry measurements, the samples were dissolved in a small amount of DMSO and after that diluted with water. The higher amount of DMSO was used to dissolve **abb**, **tbb** and molecular crystals of **2**. The crystals of **2** are more soluble in comparison with **abb** and **tbb**. The stability of coordination compounds **1** and **3** was proved by cyclic voltammetry measurements after 1, 2 and 3 days.

### 2.2. X-ray Structures of the Complexes [Ni_3_(abb)_3_(H_2_O)_3_(μ-ttc)](ClO_4_)_3_·3H_2_O·EtOH (**1**) and [(tbbH_2_)(ttcH_2_)_2_(ttcH_3_)(H_2_O)] (**2**)

**Figure 2:**
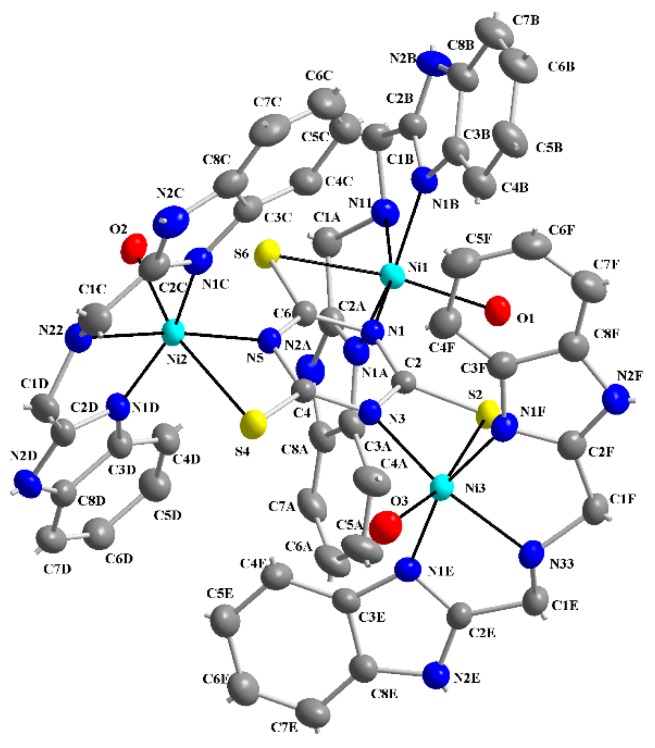
Numbering scheme of **1** with atomic displacement ellipsoids drawn at 30% probability level. Hydrogen atoms are omitted for clarity.

**Figure 3:**
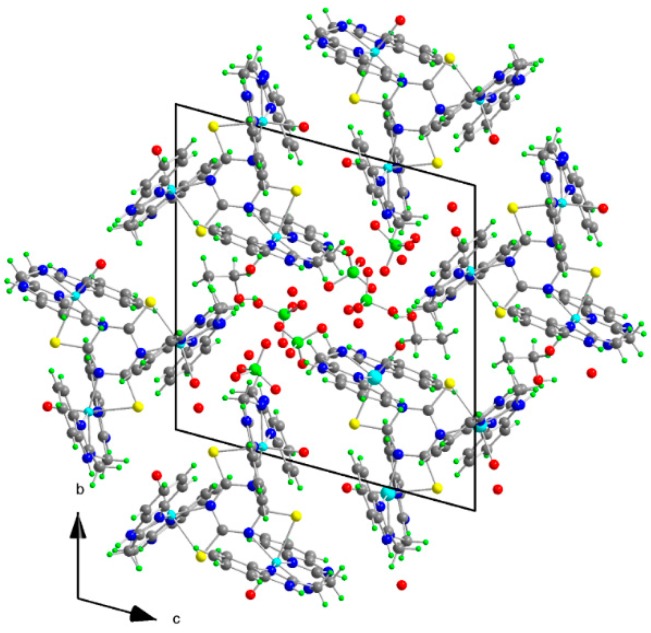
Projection of the contents of the unit cell along a-axis for **1**.

The molecular structure of **1** is depicted in Figure 2, while selected bond lengths and angles are listed in Table 1. The crystal structure (see Figure 3) is stabilized by hydrogen bonds but hydrogen atoms belonging to water molecules were not resolved. The molecular structure of **1** consists of trinuclear nickel(II) complex, ClO_4_^−^ anions, three crystal water and EtOH molecules. Three central nickel atoms are coordinated by three N atoms of **abb**, a water molecule O atom and the S,N-donor set of a **ttc** trianion in a deformed octahedral arrangement. The **ttc** trianion forms a bridge connecting the nickel centers. The bond lengths of the **abb** ring nitrogens to the central atoms are in the range of 2.041(3)–2.062(4) Å, **abb** chain Ns are in the range of 2.101(4)–2.121(3) Å and **ttc** N are in the range of 2.030(3)–2.047(3) Å, that is significantly longer bond lengths are observed for the Ni-N(**abb** chain). The Ni-S bond lengths are quite similar (2.5549(12)–2.5622(11) Å) and these lengths are much longer than Ni-S bonds found in pentacoordinated complexes [Ni(pmdien)(ttcH)] (2.3392(13) Å) [36] and [Ni_3_(pmdien)_3_(μ-ttc)](ClO_4_)_3_ (2.3821(9) Å) [33], respectively. A very complicated situation occurs in the heptanuclear complex [Ni_7_(pmdien)_6_(H_2_O)_2_(μ-ttc)_3_](ClO_4_)_5_·3H_2_O [37]. In this complex the central nickel atom is coordinated by three **ttc** anions only with Ni-S bond lengths in the 2.4663–2.4740 Å range. Two nickel atoms are hexacoordinated (three N atoms of **pmdien**, a water molecule O atom and the S,N set of **ttc**) with Ni-S lengths 2.4094 and 2.5150 Å, whereas the remaining four Ni atoms are pentacoordinated with shorter Ni-S bond lengths in the 2.3643–2.4032 Å range. A longer Ni-S bond (2.573(2) Å) was found in octahedral [Ni(dpta)(H_2_O)(ttcH)]·H_2_O, with terdentate dpta = dipropylenetriamine [38] and there is only an interaction with a Ni-S distance 2.665 Å in [Ni(taa)(ttcH)], where **taa** = tris (2-aminoethyl)amine [39].

**Table 1:** Selected bond lengths (Å) and angles (°) for **1**.

Ni1-N1B	2.041(3)	Ni2-N5	2.030(3)	Ni3-N1F	2.044(3)
Ni1-N1	2.047(3)	Ni2-N1C	2.056(4)	Ni3-N3	2.043(3)
Ni1-N1A	2.057(3)	Ni2-N1D	2.062(4)	Ni3-N1E	2.057(3)
Ni1-O1	2.102(3)	Ni2-N22	2.101(4)	Ni3-O3	2.100(3)
Ni1-N11	2.121(3)	Ni2-O2	2.109(3)	Ni3-N33	2.107(3)
N1B-Ni1-N1	101.50(14)	N5-Ni2-N1C	99.59(14)	N1F-Ni3-N3	100.24(13)
N1B-Ni1-N1A	159.97(15)	N5-Ni2-N1D	98.94(13)	N1F-Ni3-N1E	160.83(14)
N1-Ni1-N1A	97.76(13)	N1C-Ni2-N1D	161.06(14)	N3-Ni3-N1E	97.92(13)
N1B-Ni1-O1	92.65(13)	N5-Ni2-N22	170.79(14)	N1F-Ni3-O3	90.79(14)
N1-Ni1-O1	97.07(12)	N1C-Ni2-N22	80.76(15)	N3-Ni3-O3	98.86(13)
N1A-Ni1-O1	90.41(13)	N1D-Ni2-N22	80.33(14)	N1E-Ni3-O3	92.42(14)
N1B-Ni1-N11	80.27(14)	N5-Ni2-O2	96.53(12)	N1F-Ni3-N33	79.92(14)
N1-Ni1-N11	165.69(14)	N1C-Ni2-O2	90.31(13)	N3-Ni3-N33	163.52(14)
N1A-Ni1-N11	79.71(14)	N1D-Ni2-O2	91.45(13)	N1E-Ni3-N33	80.92(14)
O1-Ni1-N11	97.03(13)	N22-Ni2-O2	92.67(14)	O3-Ni3-N33	97.62(13)
N1B-Ni1-S6	89.81(10)	N5-Ni2-S4	67.54(9)	N1F-Ni3-S2	88.42(11)
N1-Ni1-S6	67.36(9)	N1C-Ni2-S4	89.22(10)	N3-Ni3-S2	67.60(9)
N1A-Ni1-S6	92.54(10)	N1D-Ni2-S4	94.26(10)	N1E-Ni3-S2	92.90(10)
O1-Ni1-S6	164.40(9)	N22-Ni2-S4	103.30(11)	O3-Ni3-S2	166.03(10)
N11-Ni1-S6	98.56(10)	O2-Ni2-S4	163.73(9)	N33-Ni3-S2	95.98(10)

**Figure 4:**
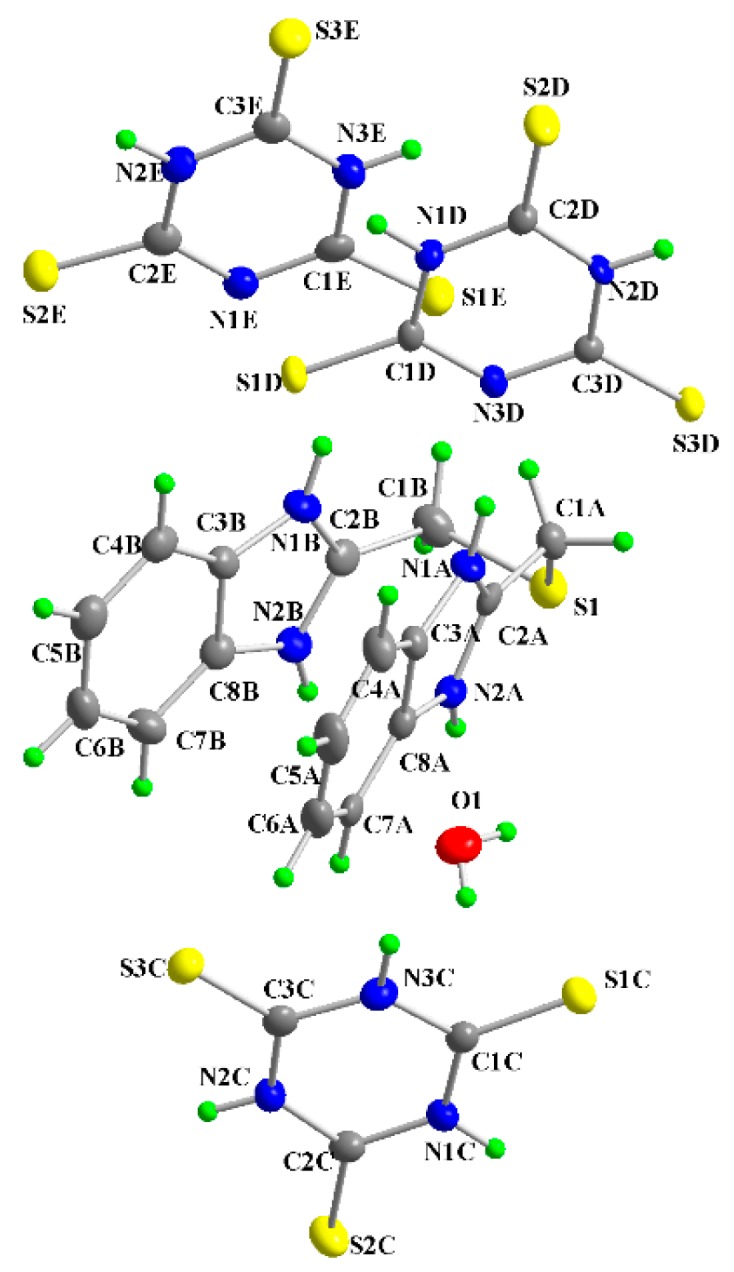
Numbering scheme of **2** with atomic displacement ellipsoids drawn at 30% probability level.

**Figure 5:**
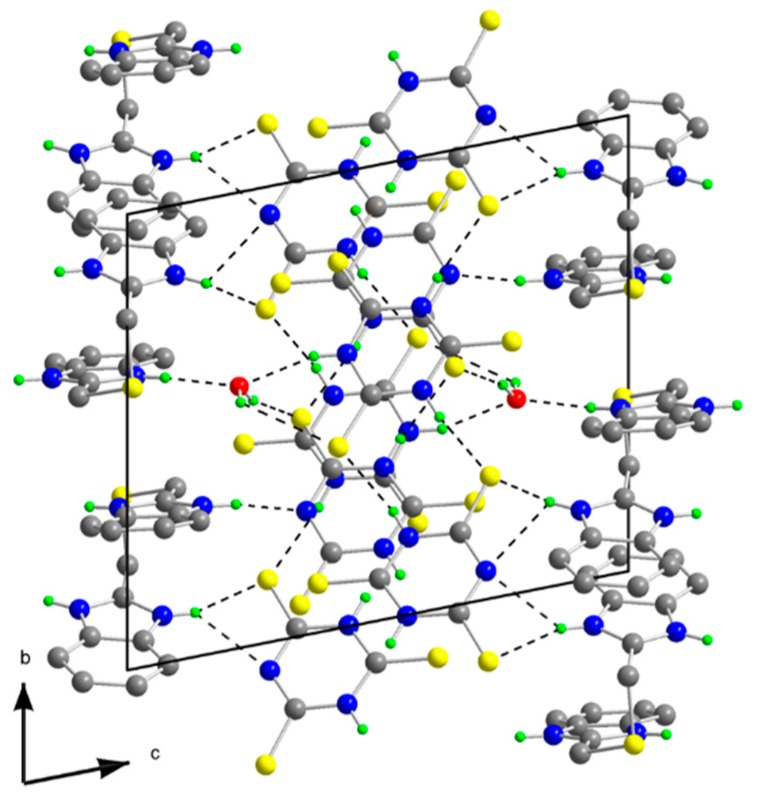
Projection of the contents of the unit cell along a-axis for **2**.

The molecular structure of [(tbbH_2_)(ttcH_2_)_2_(ttcH_3_)(H_2_O)] (**2**) is depicted in Figure 4, while selected bond lengths and angles are listed in Table 2. The structure consists from protonated **tbb**, two **ttc**H_2_ anions, **ttc**H_3_ and a water molecule. The two benzimidazole rings of **tbb** form a sandwich-like structure probably due to π-π interactions. The crystal structure is stabilized by weak hydrogen bonds (see Supplementary Tables S1 and S2, and Figure 5). The **tbb** behaves as a strong base with hydrogen atoms on all nitrogens but from the molecular structure, it is seen that in the structure there are **tbb** and **ttc** planes connected through hydrogen bonds. The S-C bond lengths (1.810 and 1.828 Å) of **tbb** are in the range typical of a single bond. The C-N bond lengths in five membered rings close to the bridge are significantly shorter (in the 1.324–1.335 Å range) than the rest of bond lengths (range of 1.389–1.405 Å). The S-C bond lengths of ttcH_3_ are in the 1.635–1.666 Å range, whereas those of **ttc**H_2_^−^ are in the 1.647–1.681 Å and 1.645–1.692 Å range, respectively. Similar S-C bond lengths were found in the structure of [Na(phen)_3_][(ttcH_2_)] [40] and [Fe(phen)_3_] (ttcH_2_)(ClO_4_) [41], where phen = 1,10-phenanthroline.

**Table 2:** Selected bond lengths (Å) and angles (°) for **2**.

S1-C1A	1.810(4)	S1C-C1C	1.666(4)	C8A-C3A-N1A	106.9(3)
S1-C1B	1.828(4)	S2C-C2C	1.635(4)	N2A-C8A-C3A	105.9(3)
C1A-C2A	1.490(5)	S3C-C3C	1.648(4)	C2B-C1B-S1	111.4(3)
N1A-C2A	1.332(5)	S1D-C1D	1.656(4)	C2B-N1B-C3B	109.4(3)
N1A-C3A	1.405(5)	S2D-C2D	1.647(4)	C2B-N2B-C8B	109.8(3)
N2A-C2A	1.335(5)	S3D-C3D	1.681(4)	N2B-C2B-N1B	109.0(3)
N2A-C8A	1.389(5)	S1E-C1E	1.675(4)	N1B-C3B-C8B	105.9(3)
C3A-C8A	1.404(5)	S2E-C2E	1.692(4)	N1C-C1C-N3C	115.0(3)
C1B-C2B	1.485(5)	S3E-C3E	1.645(4)	N2C-C2C-N1C	113.5(3)
N1B-C2B	1.331(5)	C1A-S1-C1B	101.20(18)	N2C-C3C-N3C	114.1(3)
N1B-C3B	1.397(5)	C2A-C1A-S1	115.2(3)	N1D-C2D-N2D	113.5(3)
N2B-C2B	1.324(5)	C2A-N1A-C3A	107.7(3)	N2E-C3E-N3E	113.4(3)
N2B-C8B	1.392(5)	C2A-N2A-C8A	109.1(3)		
C3B-C8B	1.397(5)	N1A-C2A-N2A	110.4(3)		

### 2.3. Biological Activity

The mechanism of the action of ligands or complexes on bacteria is very similar. When these compounds enter bacterial cells, they condense DNA to prevent DNA from replicating and cells from reproducing [42]. The basic mechanism of the action is therefore among others based on membrane protein inactivation, reduced transcription ability of mRNA into the vital cell proteins, inactivation of cytochrome b and consequent bactericidal activity [43,44].

**Figure 6:**
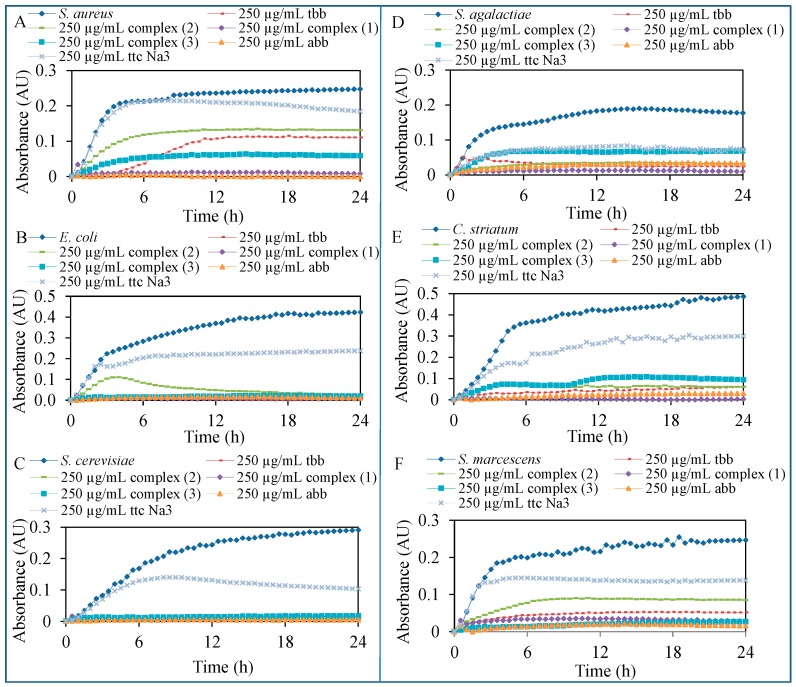
Testing of antimicrobial activity of 250 μg/mL concentration of ligands and complexes after 24 h of treatment on: (**A**) *S. aureus*; (**B**) *E. coli*; (**C**) *S. cerevisiae*; (**D**) *S. agalactiae*; (**E**) *C. striatum*; (**F**) *S. marcescens*.

The biological activity of nickel(II) perchlorate hexahydrate, nickel(II) chloride hexahydrate, the ligands **abb**, **tbb**, **ttc**Na_3_ and complexes **1**–**3** was studied. The results of the study are presented in Figure 6. The antimicrobial effect of ligands and complexes was tested on commercial *Staphylococcus aureus* (*S. aureus*), *Escherichia coli* (*E. coli*), *Saccharomyces cerevisiae* (*S. cerevisiae*) and on bacteria isolated from the infected wounds *Streptococcus agalactiae* (*S. agalactiae*), *Corynebacterium striatum* (*C. striatum*) and *Serratia marcescens* (*S. marcescens*).

The salts, ligands and complexes were applied in concentrations of 0; 3.9; 7.8; 15.6; 31.3; 62.5; 125 and 250 μg/mL on the three model organisms (representatives of Gram-negative and Gram-positive bacteria and yeasts) and the three bacterial strains isolated from the patient’s wounds and the effect was subsequently evaluated spectrophotometrically by decreasing the absorbance values in comparison with the control strains without the treatment. The highest inhibition of all tested organisms growth was observed after the treatment of ligand **abb** (*S. aureus*—97.8%, *E. coli*—94.4% and *S. cerevisiae*—86.0%, *S. agalactiae—*80.8%, *C. striatum—*93.8% and *S. marcescens—*95.5%) and complex **1** (*S. aureus*—99.2%, *E. coli—*99.8% and *S. cerevisiae*—94.0%, *S. agalactiae*—94.4%, *C. striatum—*99.6% and *S. marcescens—*88.7%), which has also in its structure the ligand **abb** (Figure 6A–F).

The eight increasing concentrations were also used to observe antimicrobial effect of each tested compounds for the statistical analysis of the viability of bacterial cells. The IC_50_ values were calculated from the absorbance measurements after the application of the concentration range in 24 h of measurements (Table 3). By these values, the previous results of antimicrobial activity were confirmed, the lowest IC_50_ values were observed in case of ligand **abb** and complex **1**. The other low values of IC_50_ were caused by uneven inhibition after the increasing concentrations of applied components. The nickel salts did not inhibit the growth of the test organisms except for *C. striatum*. The growth of this bacterium was inhibited and the IC_50_ values 16.5 and 160.3 μg/mL were calculated for nickel perchlorate and nickel chloride, respectively.

**Table 3:** Statistical calculation of IC_50_ by the STATISTICA software version 10.0 from absorbance values after the application of 0; 7.8; 15.6; 31.3; 62.5; 125 and 250 μg/mL concentrations of tested compounds.

Compounds	IC_50_ (μg/mL) Measurement after 24 h					
	*S. aureus*	*E. coli*	*S. cerevisiae*	*S. agalactiae*	*C. striatum*	*S. marcescens*
Ligand **abb**	5.6	19	21.4	22.4	30.8	26.7
Ligand **tbb**	485.2	182.3	204.7	126.1	112.5	122.6
Ligand **ttc**Na_3_	625	636.8	86.4	119.5	115.8	123.9
Complex **1**	4.8	23.7	15.4	21.4	24.3	27.2
Complex **2**	562.3	257.6	64.3	102.1	119.5	61.5
Complex **3**	1036.7	12.6	18.2	113.4	124.9	110.4

The highest antimicrobial activity on all studied microorganisms was observed for **abb** and complex **1**. The IC_50_ values are in these cases comparable although the synergistic effect of nickel and ligands in **1** must play an important role when we take in to account the amount of **abb** in the tested samples. When we compare the activities of **abb** and **tbb**, which only differ in the chain atoms in the middle (N or S), the activity of **tbb** is very low. The combination of **tbb** and **ttc** in complex **2** leads to a better activity against *S. cerevisiae* only, but the sodium salt of trithiocyanuric acid shows similar activity. The coordination compound **3** was prepared to compare the effect of ligands **pmdien** and **abb**, which are coordinated to central nickel atoms. The trinuclear complexes **1** and **3** are structurally very similar although nickel atoms in **1** are octahedral due to the presence of coordinated water molecules whereas the bulky **pmdien** ligand in **3** does not allow coordination of water and the nickel atoms are pentacoordinated. These changes in coordination modes and ligands also lead to different biological properties of the complexes. Complex **1** is active against all the treated microorganisms whereas complex **3** shows no activity against *S. aureus*, but it shows better activity against *E. coli* and similar activity against *S. cerevisiae* in comparison with **1**.

## 3. Experimental Section

### 3.1. General Information

*Safety note*: *Caution!* Perchlorate salts of metal complexes with organic ligands are potentially explosive and should be handled with great care.

The chemicals and solvents were supplied by Aldrich (St. Louis, MO, USA) and used without further purification. The C, H, N, and S analyses were carried out on an EA 1108 instrument (Fisons Instruments, Rodano, Italy). IR spectra (400–4000 cm^−1^) were recorded with the help of a Tensor 27 FTIR spectrometer (Bruker, Billerica, MA, USA) using KBr pellets. The absorption spectra in MetOH (230–1100 nm) were obtained on a Specord 210 UV/VIS spectrometer (Analytik Jena AG, Jena, Germany). The crystallographic data for the structures **1** and **2** has been deposited with the Cambridge Crystallographic Data Centre as supplementary publications No. 1033059 and 1033060. Copies of the data can be obtained, free of charge, on application to CCDC, 12 Union Road, Cambridge, CB2 1EZ, UK, (Fax: +44-0-1223-336033 or E-Mail: deposit@ccdc.cam.ac.uk).

### 3.2. Preparation of the Compounds

#### 3.2.1. 1-(1*H*-Benzimidazol-2-yl)-*N*-(1*H*-benzimidazol-2-ylmethyl)methanamine (**abb**)

The ligand was prepared according to Matthews *et al.* [45]. Iminodiacetic acid (6.65 g, 50 mmol) and *o*-phenylenediamine (10.82 g, 100 mmol) were mixed in a solution of 18% HCl (100 mL). The solution was heated under reflux for 3 days. A green precipitate formed after cooling was dissolved after the addition of water (300 mL). The solution was stirred and ammonia (28%) was added until the pH of the solution became 9. During the addition of ammonia, a light brown precipitate was obtained. The precipitate was collected on a frit, washed several times with water and dried at 40 °C. Yield: 32.3%. *Anal*. Calcd. for **abb**·4H_2_O: C, 55.0; H, 6.6; N, 20.0; S, 0.0. Found: C, 55.0; H, 6.6; N, 19.5; S, 0.0%. IR (cm^−1^): 405w, 447w, 480w, 533w, 742vs, 767m, 876m, 1015m, 1110m, 1222m, 1273vs, 1310w, 1331m, 1438vs, 1454s, 1532w, 1623m, 2851m, 3055s, 3226s, 3368m. UV-Vis (MeOH/nm): 324, 378, 428.

#### 3.2.2. 2-(1*H*-Benzimidazol-2-ylmethylsulfanylmethyl)-1*H*-benzimidazole (**tbb**)

The ligand was prepared according to Matthews *et al.* [45]. Thiodiacetic acid (15.02 g, 100 mmol) and *o*-phenylenediamine (21.63 g, 200 mmol) were mixed in a solution of 18% HCl (230 mL). The solution was heated under reflux 3 d. A green precipitate formed after cooling was dissolved after addition of water (500 mL). The solution was stirred and ammonia (28%) was added until the pH of the solution became 9. During the addition of ammonia, a light brown precipitate was obtained. The precipitate was collected on a frit, washed several times with water and dried at 40 °C. Yield: 32.3%. *Anal*. Calcd. for **tbb**·3/2H_2_O: C, 59.8; H, 5.3; N, 17.4; S, 10.0. Found: C, 60.1; H, 5.2; N, 17.2; S, 9.8%. IR (cm^−1^): 437w, 736vs, 844w, 1027m, 1129w, 1159m, 1226m, 1273s, 1313m, 1440vs, 1454s, 1535m, 1625w, 2784m, 2939s, 3059s, 3377m. UV-Vis (MeOH/nm): 290, 322, 340.

#### 3.2.3. [Ni_3_(abb)_3_(H_2_O)_3_(μ-ttc)](ClO_4_)_3_·3H_2_O·EtOH (**1**)

The ligand **abb** (0.35 g, 1 mmol) was dissolved in EtOH (100 mL) and added with stirring to a solution of nickel(II) perchlorate hexahydrate (0.36 g, 1 mmol) in 10 mL of EtOH. This red solution was heated to boiling and after cooling **ttc**Na_3_·9H_2_O (0.13 g, 0.33 mmol) in water: EtOH (1:1) mixture was added with stirring. A light red solution was left for crystallization. After a week, grey green plate crystals suitable for X-ray analysis were collected. Yield: 64%. *Anal*. Calcd.: C, 38.9; H, 3.9; N, 15.4; S, 5.9. Found: C, 38.7; H, 3.5; N, 15.6; S, 5.7%. IR (cm^−1^): 430w, 627s, 764s, 913m, 1088vs, 1111vs, 1144s, 1239m, 1322w, 1338w, 1437s, 1454s, 1542w, 1624m, 2918m, 3064br, 3193br, 3381s. UV-Vis (MeOH/nm): 371, 492, 541, 585, 801, 997.

#### 3.2.4. [(tbbH_2_)(ttcH_2_)_2_(ttcH_3_)(H_2_O)] (**2**)

The crystals of **2** were prepared by mixing **tbb** and **ttc**H_3_ ethanol solutions in 1:3 molar ratio. Yellow needle-like crystals suitable for X-ray analysis were obtained after a week. Yield: 75%. Anal. Calcd.: C, 35.6; H, 3.0; N, 21.6; S, 38.0. Found: C, 35.4; H, 3.1; N, 21.3; S, 37.7%. IR (cm^−1^): 457m, 665w, 746w, 782w, 1120vs, 1258w, 1296w, 1360s, 1541vs, 1574s, 1715w, 2522w, 2907m, 3039m, 3142s, 3444br. UV-Vis (MeOH/nm): 300, 341.

#### 3.2.5. [Ni_3_(pmdien)_3_(μ-ttc)](ClO_4_)_3_ (**3**)

The complex [Ni_3_(pmdien)_3_(μ-ttc)](ClO_4_)_3_, where **pmdien** = *N*,*N*,*N*′,*N*′′,*N*′′-pentamethyldiethylene-triamine, was prepared according to Kopel *et al.* [33], by reaction of **pmdien** (0.2 mL, 1 mmol) with Ni(ClO_4_)_2_·6H_2_O (0.37 g, 1 mmol) in EtOH, followed by the addition of a solution of **ttc**Na_3_·9H_2_O (0.14 g, 0.33 mmol). Yield: 72%. Anal. Calcd.: C, 30.8; H, 6.0; N, 14.4; S, 8.2. Found: C, 30.4; H, 5.9; N, 14.1; S, 7.7%.

### 3.3. X-ray Crystallography

X-ray data of **1** and **2** were collected on a SMART CCD diffractometer (Siemens, Madison, WI, USA) with Mo-Kα radiation (λ = 0.71073 Å, graphite monochromator). The crystal was cooled to 173(2) K by a flow of nitrogen gas using the LT-2A device. A full sphere of reciprocal space was scanned by 0.3 steps in ω with a crystal-to-detector distance of 3.97 cm. Preliminary orientation matrices were obtained from the first frames using SMART [46]. The collected frames were integrated using the preliminary orientation matrix which was updated every 100 frames. Final cell parameters were obtained by refinement of the positions of reflections with I > 10σ (I) after integration of all the frames using SAINT software [46]. The data were empirically corrected for absorption and other effects using the SADABS program [47]. The structures were solved by direct methods and refined by full-matrix least squares on all |*F*^2^| data using SHELXTL software [48].

X-ray data of **1**: Important crystallographic parameters are as follows: C_53_H_48_Cl_3_N_18_O_19_S_3_Ni_3_, wavelength 0.71073 Å, triclinic, space group Pī, a = 14.7409(7), b = 16.2290(8), c = 16.3145(8) Å, α = 103.367(1)°, β = 108.538(1)°, γ = 93.087(1)°, volume 3565.7(3) Å^3^, Z = 2, density (calc.) 1.509 Mg/m^3^, absorption coefficient 1.063 mm^−1^, F(000) = 1654, crystal size 0.42 mm × 0.32 mm × 0.08 mm, index ranges −19 ≤ *h* ≤ 19, −21 ≤ *k* ≤ 21, −22 ≤ *l* ≤ 22, reflections collected/independent 51144/18385 (*R*_int_ = 0.0648), data/restraints/parameters 18385/0/894, goodness-of fit on *F*^2^ = 1.027, final *R*_1_ (I > 2σ(I) data) = 0.0713, *wR*_2_ = 0.2059, final *R*_1_ (all data) = 0.0986, *wR*_2_ = 0.2259. The largest peak and hole on the final difference map were 2.900 and −1.379 e.Å^−3^.

X-ray data of **2**: Important crystallographic parameters are as follows: C_25_H_25_N_13_OS_10_, wavelength 0.71073 Å, triclinic, space group Pī, a = 11.2463(10), b = 13.0425(12), c = 13.6109(13) Å, α = 74.757(2)°, β = 77.406(2)°, γ = 65.299(2)°, volume 1736.6(3) Å^3^, Z = 2, density (calc.) 1.614 Mg/m^3^, absorption coefficient 0.681 mm^−1^, F(000) = 868, crystal size 0.44 mm × 0.08 mm × 0.02 mm, index ranges −13 ≤ *h* ≤ 13, −15 ≤ *k* ≤ 15, −16 ≤ *l* ≤ 16, reflections collected/independent 18662/6149 (*R*_int_ = 0.0682), data/restraints/parameters 6149/0/448, goodness-of fit on *F*^2^ = 1.018, final *R*_1_ (I > 2σ(I) data) = 0.0558, *wR*_2_ = 0.1327, final *R*_1_ (all data) = 0.0832, *wR*_2_ = 0.1443. The largest peak and hole on the final difference map were 0.382 and −0.571 e.Å^−3^.

### 3.4. Biological Activity Testing

#### 3.4.1. Cultivation of Commercially Supplied Bacteria and Yeasts

*Staphylococcus aureus* (NCTC 8511), *Escherichia coli* (NCTC 13216) and *Saccharomyces cerevisiae* (ATCC 9763) were obtained from the Czech Collection of Microorganisms, Faculty of Science, Masaryk University (Brno, Czech Republic). The strains were stored as a spore suspension in 20% (*v*/*v*) glycerol at −20 °C. Prior to use in this study, the strains were thawed and the glycerol was removed by washing with distilled water. The composition of cultivation medium was as follows: glucose 10 g/L, tryptone 10 g/L and yeast extract 5 g/L, sterilized MilliQ water with 18 MΩ. pH of the cultivation medium was adjusted at 7.4 before sterilization. The sterilization of the media was carried out at 121 °C for 30 min in sterilizer (Tuttnauer 2450EL, Beit Shemesh, Israel). The prepared cultivation media were inoculated with bacterial cultures or yeasts into 25 mL Erlenmeyer flasks. After the inoculation, bacteria and yeasts were cultivated for 24 h on a shaker at 600 rpm and 37 °C. The strains, cultivated under these conditions, were diluted by cultivation medium to OD_600_ = 0.1 and used in the following experiments.

#### 3.4.2. Preparation of Hospital Samples and Their Cultivation

##### Cohort of Patients with Bacterial Infections

For the evaluation, patients with surface or deep infection severity were selected. The patients were with the age between 19 and 93 years. Enrollment of the patients into the clinical study was approved by the Ethics Committee of Trauma Hospital in Brno.

##### Collection of Wound Swabs from Patients with Bacterial Infections

Smears were collected from the infected wounds with the agreement of the patients. The smear was sampled by a rolling motion at the site of skin puncture using a sterile swab sampler. Subsequently, the swab was removed into a tube with a semi-solid transport medium (inorganic salts, sodium thioglycolate, 1% agar, activated charcoal) and carefully sealed and labeled. The marked tubes were immediately examined microbiologically.

##### Cultivation of Clinical Specimens

The isolation of bacterial strains was performed using selective blood agar. The swab samples were cultivated on blood agar with 10% of NaCl, blood agar with no other compounds [49], Endo agar [50] and blood agar with amikacin [51]. These Petri dishes were cultivated for 24–48 h at 37 °C. For the cultivation of all bacterial specimens TGY medium (glucose 1 g/L, tryptone 5 g/L, yeast extracts 2.5 g/L) was used.

#### 3.4.3. Determination of Growth Curves

The procedure for the evaluation of the antimicrobial effect of tested compounds consisted in measuring of the absorbance using the apparatus Multiskan EX (Thermo Fisher Scientific, Vantaa, Finland) and subsequent analysis in the form of growth curves. Bacteria and yeasts were cultivated in GTY medium for 24 h with shaking and were diluted with GTY medium to absorbance 0.1 before analysis using a Specord spectrophotometer 210 at a wavelength of 600 nm. On the microplate, these cultures were mixed with various concentrations of four types of antibiotics (ampicillin, streptomycin, penicillin and tetracycline) or *S. aureus* alone as a control for the measurements. The concentrations of antibiotics were 0, 7.8, 15.6, 31.3, 62.5, 125 and 250 μg/mL. The total volume in the microplate wells was always 300 μL. The measurements were carried out at time 0, then each half-hourly for 24 h at 37 °C and a wavelength of 600 nm. The obtained values were analysed in a graphical form as growth curves for each variant individually.

#### 3.4.4. Statistical Analyses

The software STATISTICA (data analysis software system), version 10.0 (Statsoft Inc. ed., Tulsa, OK, USA) was used for the data processing. The half-maximal concentrations (IC_50_) were calculated from logarithmic regression of sigmoidal dose-response curve. The general regression model was used to analyse differences between the combinations of the compounds.

## 4. Conclusions

In this work we present our results for bis(benzimidazole) and trithiocyanurate complexes and their use as antimicrobial agents in the treatment of bacterial infections. For these purposes bis(benzimidazoles) were synthesized and then further used for the preparation of coordination compound and molecular crystals. The X-ray single crystal analysis proved the structures of trinuclear Ni(II) complex and bis(benzimidazole)‒trithiocyanurate co-crystals. It was shown that these compounds are biologically active. Especially the ligand **abb** and the complex **1**, where the ligand is coordinated, showed very good activities against the wide spectrum of commercially available bacterial and yeast strains (*S. aureus*, *E. coli* and *S. cerevisiae*) and clinical specimens from real patients with non-healing infections and complicated courses of treatment.

## Supplementary Materials

Supplementary materials can be accessed at: http://www.mdpi.com/1420-3049/20/06/10360/s1.

## References

[1] Giovine A., Muraglia M., Florio M.A., Rosato A., Corbo F., Franchini C., Degennaro L., Musio B., Luisi R. (2014). Synthesis of Functionalized Arylaziridines as Potential Antimicrobial Agents. Molecules.

[2] Ranjith P.K., Rajeesh P., Haridas K.R., Susanta N.K., Row T.N.G., Rishikesan R., Kumari N.S. (2013). Design and synthesis of positional isomers of 5 and 6-bromo-1-(phenyl)sulfonyl-2-(4-nitrophenoxy)methyl-1*H*-benzimidazoles as possible antimicrobial and antitubercular agents. Bioorg. Med. Chem. Lett..

[3] Toro P., Klahn A.H., Pradines B., Lahoz F., Pascual A., Biot C., Arancibia R. (2013). Organometallic benzimidazoles: Synthesis, characterization and antimalarial activity. Inorg. Chem. Commun..

[4] Sondhi S.M., Rajvanshi S., Johar M., Bharti N., Azam A., Singh A.K. (2002). Anti-inflammatory, analgesic and antiamoebic activity evaluation of pyrimido 1,6-alpha benzimidazole derivatives synthesized by the reaction of ketoisothiocyanates with mono and diamines. Eur. J. Med. Chem..

[5] Grassmann S., Sadek B., Ligneau X., Elz S., Ganellin C.R., Arrang J.M., Schwartz J.C., Stark H., Schunack W. (2002). Progress in the proxifan class: Heterocyclic congeners as novel potent and selective histamine H_3_-receptor antagonists. Eur. J. Pharm. Sci..

[6] Haque R.A., Iqbal M.A., Asekunowo P., Majid A., Ahamed M.B.K., Umar M.I., Al-Rawi S.S., Al-Suede F.S.R. (2013). Synthesis, structure, anticancer, and antioxidant activity of para-xylyl linked bis-benzimidazolium salts and respective dinuclear Ag(I) *N*-heterocyclic carbene complexes (Part-II). Med. Chem. Res..

[7] Nile S.H., Kumar B., Park S.W. (2013). *In Vitro* Evaluation of Selected Benzimidazole Derivatives as an Antioxidant and Xanthine Oxidase Inhibitors. Chem. Biol. Drug Des..

[8] Kralova V., Hanusova V., Stankova P., Knoppova K., Canova K., Skalova L. (2013). Antiproliferative effect of benzimidazole anthelmintics albendazole, ricobendazole, and flubendazole in intestinal cancer cell lines. Anti-Cancer Drugs.

[9] Jain A., Sharma R., Chaturvedi S.C. (2013). A rational design, synthesis, characterization, and antihypertensive activities of some new substituted benzimidazoles. Med. Chem. Res..

[10] Beaulieu C., Wang Z.Y., Denis D., Greig G., Lamontagne S., O’Neill G., Slipetz D., Wang J. (2004). Benzimidazoles as new potent and selective DP antagonists for the treatment of allergic rhinitis. Bioorg. Med. Chem. Lett..

[11] White A.W., Curtin N.J., Eastman B.W., Golding B.T., Hostomsky Z., Kyle S., Maegley K.A., Li J., Skalitzky D.J., Webber S.E. (2004). Potentiation of cytotoxic drug activity in human tumour cell lines, by amine-substituted 2-arylbenzimidazole-4-carboxamide PARP-1 inhibitors. Bioorg. Med. Chem. Lett..

[12] Kahveci B., Mentes E., Ozil M., Ulker S., Erturk M. (2013). An efficient synthesis of benzimidazoles via a microwave technique and evaluation of their biological activities. Mon. Chem..

[13] Purushottamachar P., Godbole A.M., Gediya L.K., Martin M.S., Vasaitis T.S., Kwegyir-Afful A.K., Ramalingam S., Ates-Alagoz Z., Njar V.C.O. (2013). Systematic Structure Modifications of Multitarget Prostate Cancer Drug Candidate Galeterone To Produce Novel Androgen Receptor Down-Regulating Agents as an Approach to Treatment of Advanced Prostate Cancer. J. Med. Chem..

[14] Evans T.M., Gardiner J.M., Mahmood N., Smis M. (1997). Structure-activity relationships of anti-HIV-1 *N*-alkoxy- and *N*-allyloxy-benzimidazoles. Bioorg. Med. Chem. Lett..

[15] Yoon Y.K., Ali M.A., Wei A.C., Choon T.S., Khaw K.Y., Murugaiyah V., Masand V.H., Osman H. (2013). Synthesis, characterization, and molecular docking analysis of novel benzimidazole derivatives as cholinesterase inhibitors. Bioorg. Chem..

[16] Zhu J.M., Wu C.F., Li X.B., Wu G.S., Xie S., Hu Q.N., Deng Z.X., Zhu M.X., Hong X.C., Luo H.R. (2013). Synthesis, biological evaluation and molecular modeling of substituted 2-aminobenzimidazoles as novel inhibitors of acetylcholinesterase and butyrylcholinesterase. Bioorg. Med. Chem..

[17] Ceballos L., Virkel G., Elissondo C., Canton C., Canevari J., Murno G., Denegri G., Lanusse C., Alvarez L. (2013). A pharmacology-based comparison of the activity of albendazole and flubendazole against Echinococcus granulosus metacestode in sheep. Acta Trop..

[18] Perez-Villanueva J., Hernandez-Campos A., Yepez-Mulia L., Mendez-Cuesta C., Mendez-Lucio O., Hernandez-Luis F., Castillo R. (2013). Synthesis and antiprotozoal activity of novel 2-[2-(1*H*-imidazol-1-yl)ethyl sulfanyl]-1*H*-benzimidazole derivatives. Bioorg. Med. Chem. Lett..

[19] Henke K.R., Robertson D., Krepps M.K., Atwood D.A. (2000). Chemistry and stability of precipitates from aqueous solutions of 2,4,6-trimercaptotriazine, trisodium salt, nonahydrate (TMT-55) and mercury (II) chloride. Water Res..

[20] Matlock M.M., Henke K.R., Atwood D.A., Robertson D. (2001). Aqueous leaching properties and environmental implications of cadmium, lead and zinc trimercaptotriazine (TMT) compounds. Water Res..

[21] Rosso V.W., Lust D.A., Bernot P.J., Grosso J.A., Modi S.P., Rusowicz A., Sedergran T.C., Simpson J.H., Srivastava S.K., Humora M.J. (1997). Removal of palladium from organic reaction mixtures by trimercaptotriazine. Org. Process Res. Dev..

[22] Garrett C.E., Prasad K. (2004). The art of meeting palladium specifications in active pharmaceutical ingredients produced by Pd-catalyzed reactions. Adv. Synth. Catal..

[23] Chen W., Hong S., Xiang B., Luo H.Q., Li M., Li N.B. (2013). Corrosion inhibition of copper in hydrochloric acid by coverage with trithiocyanuric acid self-assembled monolayers. Corros. Eng. Sci. Technol..

[24] Chen W., Hong S., Luo H.Q., Li N.B. (2014). Inhibition Effect of 2,4,6-Trimercapto-1,3,5-triazine Self-Assembled Monolayers on Copper Corrosion in NaCl Solution. J. Mater. Eng. Perform..

[25] Iltzsch M.H., Tankersley K.O. (1994). Structure-activity relationship of ligands of uracil phosphoribosyltransferase from toxoplasma-gondii. Biochem. Pharmacol..

[26] Kar S., Miller T.A., Chakraborty S., Sarkar B., Pradhan B., Sinha R.K., Kundu T., Ward M.D., Lahiri G.K. (2003). Synthesis, mixed valence aspects and non-linear optical properties of the triruthenium complexes [{(bpy)_2_Ru^II^}_3_(L)]^3+^ and [{(phen)_2_Ru^II^}_3_L^3+^ (bpy = 2,2′-bipyridine, phen = 1,10-phenanthroline and L^3−^ = 1,3,5-triazine-2,4,6-trithiol). Dalton Trans..

[27] Kar S., Pradhan B., Sinha K., Kundu T., Kodgire P., Rao K.K., Puranik V.G., Lahiri G.K. (2004). Synthesis, structure, redox, NLO and DNA interaction aspects of [{(L′^–^′′′)_2_Ru^II^}_3_(mu_3_-L)]^3+^ and [(L′)_2_Ru^II^(NC_5_H_4_S^−^)]^+^, L^3−^ = 1,3,5-triazine-2,4,6-trithiolato, L′^–^′′′ = arylazopyridine. Dalton Trans..

[28] Aoki S., Zulkefeli M., Shiro M., Kimura E. (2002). New supramolecular trigonal prisms from zinc(II)-1,4,7,10-tetraazacyclododecane (cyclen) complexes and trithiocyanurate in aqueous solution. Proc. Natl. Acad. Sci. USA.

[29] Aoki S., Shiro M., Kimura E. (2002). A cuboctahedral supramolecular capsule by 4:4 self-assembly of tris(Zn^II^-cyclen) and trianionic trithiocyanurate in aqueous solution at neutral pH (cyclen = 1,4,7,10-tetraazacyclododecane). Chem. Eur. J..

[30] Zulkefeli M., Sogon T., Takeda K., Kimura E., Aoki S. (2009). Design and Synthesis of a Stable Supramolecular Trigonal Prism Formed by the Self-Assembly of a Linear Tetrakis(Zn^2+^-cyclen) Complex and Trianionic Trithiocyanuric Acid in Aqueous Solution and Its Complexation with DNA (Cyclen = 1,4,7,10-Tetraazacyclododecane). Inorg. Chem..

[31] Kopel P., Dolezal K., Machala L., Langer V. (2007). Synthesis, characterization and screening of biological activity of Zn(II), Fe(II) and Mn(II) complexes with trithiocyanuric acid. Polyhedron.

[32] Kopel P., Dolezal K., Langer V., Jun D., Adam V., Kuca K., Kizek R. (2014). Trithiocyanurate Complexes of Iron, Manganese and Nickel and Their Anticholinesterase Activity. Molecules.

[33] Kopel P., Mrozinski J., Dolezal K., Langer V., Boca R., Bienko A., Pochaba A. (2009). Ferromagnetic Properties of a Trinuclear Nickel(II) Complex with a Trithiocyanurate Bridge. Eur. J. Inorg. Chem..

[34] Ranganathan A., Pedireddi V.R., Rao C.N.R. (1999). Hydrothermal synthesis of organic channel structures: 1:1 Hydrogen-bonded adducts of melamine with cyanuric and trithiocyanuric acids. J. Am. Chem. Soc..

[35] Nagarajan V., Pedireddi V.R. (2014). Preparation of Multiple Cocrystals of Trithiocyanuric Acid with Some *N*-Donor Compounds. Cryst. Growth Des..

[36] Kopel P., Travnicek Z., Kvitek L., Biler M., Pavlicek M., Sindelar Z., Marek J. (2001). Coordination compounds of nickel with trithiocyanuric acid. Part IV. Structure of Ni(pmdien)(ttcH) (pmdien = *N*,*N*,*N*′,*N*′,*N*′′-pentamethyldiethylenetriamine, ttcH_3_ = trithiocyanuric acid). Transit. Met. Chem..

[37] Bienko A., Kopel P., Kizek R., Kruszynski R., Bienko D., Titis J., Boca R. (2014). Synthesis, crystal structure and magnetic properties of trithiocyanurate or thiodiacetate polynuclear Ni(II) and Co(II) complexes. Inorg. Chim. Acta.

[38] Kopel P., Travnicek Z., Panchartkova R., Sindelar Z., Marek J. (1998). Coordination compounds of nickel with trithiocyanuric acid. J. Coord. Chem..

[39] Kopel P., Travnicek Z., Panchartkova R., Biler M., Marek J. (1999). Coordination compounds of nickel with trithiocyanuric acid. Part II. Crystal and molecular structure of Ni(taa)(ttcH) (taa = tris-(2-aminoethyl)amine, ttcH_3_ = trithiocyanuric acid). Transit. Met. Chem..

[40] Marek J., Kopel P., Travnicek Z. (2003). Tris(1,10-phenanthroline)sodium 2,4,6-trimercapto-1,3,5-triazin-1-ide. Acta Crystallogr. Sect. C Cryst. Struct. Commun..

[41] Kopel P., Travnicek Z., Zboril R., Marek J. (2004). Synthesis, X-ray and Mossbauer study of iron(II) complexes with trithiocyanuric acid (ttcH_3_): The X-ray structures of [Fe(bpy)_3_) (ttcH) 2bpy 7H_2_O and [Fe(phen)_3_](ttcH_2_)(ClO_4_) 2CH_3_OH 2H_2_O. Polyhedron.

[42] Li W.R., Xie X.B., Shi Q.S., Duan S.S., Ouyang Y.S., Chen Y.B. (2011). Antibacterial effect of silver nanoparticles on Staphylococcus aureus. Biometals.

[43] Park H.J., Kim J.Y., Kim J., Lee J.H., Hahn J.S., Gu M.B., Yoon J. (2009). Silver-ion-mediated reactive oxygen species generation affecting bactericidal activity. Water Res..

[44] Kwakye-Awuah B., Williams C., Kenward M.A., Radecka I. (2008). Antimicrobial action and efficiency of silver-loaded zeolite X. J. Appl. Microbiol..

[45] Matthews C.J., Leese T.A., Clegg W., Elsegood M.R.J., Horsburgh L., Lockhart J.C. (1996). A route to bis(benzimidazole) ligands with built-in asymmetry: Potential models of protein binding sites having histidines of different basicity. Inorg. Chem..

[46] (2003). SMART (Version 5.63) and SAINT (Version 6.45), Area Detector Control and Integration Software.

[47] Sheldrick G.M. (2003). SADABS Program for Empirical Absorption Correction for Area Detectors, Version 2.10.

[48] Sheldrick G.M. (2008). A short history of SHELX. Acta Crystallogr. Sect. A.

[49] Bautista-Trujillo G.U., Solorio-Rivera J.L., Renteria-Solorzano I., Carranza-German S.I., Bustos-Martinez J.A., Arteaga-Garibay R.I., Baizabal-Aguirre V.M., Cajero-Juarez M., Bravo-Patino A., Valdez-Alarcon J.J. (2013). Performance of culture media for the isolation and identification of Staphylococcus aureus from bovine mastitis. J. Med. Microbiol..

[50] Predrag S., Branislava K., Miodrag S., Biljana M.S., Suzana T., Natasa M.T., Tatjana B. (2012). Clinical importance and representation of toxigenic and non-toxigenic Clostridium difficile cultivated from stool samples of hospitalized patients. Braz. J. Microbiol..

[51] Bosch-Mestres J., Martin-Fernandez R.M., de Anta-Losada M.T.J. (2003). Comparative study of three culture media for detecting group B Streptococcus colonization in pregnant women. Enferm. Infec. Microbiol. Clin..

