# Supplementary material for: Biological Activity and Molecular Structures of Bis(benzimidazole) and Trithiocyanurate Complexes
Source: Molecules. 2015 Jun 4;20(6):10360–76. doi: 10.3390/molecules200610360 (PMC6272323; doi:10.3390/molecules200610360)
Supplement: Supplementary file 1 [file molecules-20-10360-s001.pdf]

Supplementary Materials

Table S1. Hydrogen bonds for **1** (Å, °).

**D-H...A**	**d(D-H)**	**d(H...A)**	**d(D...A)**	**<(DHA)**
N2A-H2A...O34 ^{i}	0.88	2.19	2.958(5)	146
N2A-H2A...O2 ^{ii}	0.88	2.37	3.079(5)	138
N2B-H2B...O1S	0.88	1.96	2.811(5)	161
N2C-H2C...O33 ^{iii}	0.88	2.13	2.890(5)	145
N2D-H2D...O14 ^{iv}	0.88	2.02	2.894(6)	177
N2E-H2E...O24 ^{v}	0.88	2.03	2.893(5)	166
N2F-H2F...O2W	0.88	1.96	2.818(6)	164
O1S-H1S...O1W	0.84	1.92	2.740(7)	165

Symmetry transformations used to generate equivalent atoms: (i): x, y, z + 1; (ii): −x + 1, −y + 1, −z + 2; (iii): x − 1, y, z; (iv): −x + 1, −y + 1, −z + 1; (v): −x + 1, −y, −z + 1.

Table S2. Hydrogen bonds for **2** (Å, °).

**D-H...A**	**d(D-H)**	**d(H...A)**	**d(D...A)**	**<(DHA)**
N1A-H1A...N3D	0.88	1.85	2.719(4)	169
N2A-H2A...O1	0.88	1.97	2.818(4)	160
N1B-H1B...N1E	0.88	2.46	3.160(4)	136
N1B-H1B...S1E	0.88	2.48	3.295(3)	154
N2B-H2B...S2E ^{i}	0.88	2.48	3.274(3)	151
N2B-H2B...N1E ^{i}	0.88	2.57	3.125(4)	122
N1C-H1C...S2E ^{ii}	0.88	2.42	3.273(3)	164
N2C-H2C...S1E ^{iii}	0.88	2.40	3.274(3)	172
N3C-H3C...O1	0.88	2.10	2.952(4)	162
N1D-H1D...S1D ^{iv}	0.88	2.49	3.359(3)	168
N2D-H2D...S3D ^{v}	0.88	2.43	3.306(3)	174
N2E-H2E...S1C ^{vi}	0.88	2.42	3.287(3)	170
N3E-H3E...S3C ^{vii}	0.88	2.45	3.330(3)	175
O1-H1...S3D ^{viii}	0.80(5)	2.53(5)	3.302(3)	164
O1-H2...S1C	0.83(5)	2.88(5)	3.344(3)	117

Symmetry transformations used to generate equivalent atoms: (i): −x, −y + 2, −z + 2; (ii): x, y − 1, z + 1; (iii): x, y, z + 1; (iv): −x + 1, −y + 2, −z + 1; (v): −x + 1, −y + 1, −z + 1; (vi): x, y + 1, z – 1; (vii): x, y, z – 1; (viii): −x + 1, −y + 1, −z + 2.

Electrochemical behaviour of the prepared complexes was studied in water solution using cyclic voltammetry. Electrochemical measurements were performed with an AUTOLAB Analyzer (Metrohm, Herisau, Switzerland) connected to a VA-Stand 663 (Metrohm), using a standard cell with three electrodes. The three-electrode cell used a reference Li^+/Li electrode (E0 of the ferrocene/ferrocenium couple +470 mV) with a Luggine capillary and a Pt wire sealed in glass (0.5 mm diameter, 0.00196 cm^2). For smoothing and baseline correction the software GPES 4.9 supplied by EcoChemie (Utrecht, Netherlands) was employed. The analysed samples were deoxygenated prior to measurements by purging with argon (99.999%) for 120 s. All experiments were carried out at room temperature 20 °C. Tetramethylammonium perchlorate (TMAP, 0.1 M) was used as supporting electrolyte and the solutions were 1×10^{-3} M in complex. All the potentials are referred against

reference electrode used in this work. The CV studies were also performed in the presence of serum as a function of time and also in a growth medium which contained 10 g/L of glucose, 10 g/L of tryptone (mixture of aminoacids) and 5 g/L of yeast extract at pH 7. The result of the study shows on the stability of the complexes (see Figure S1).

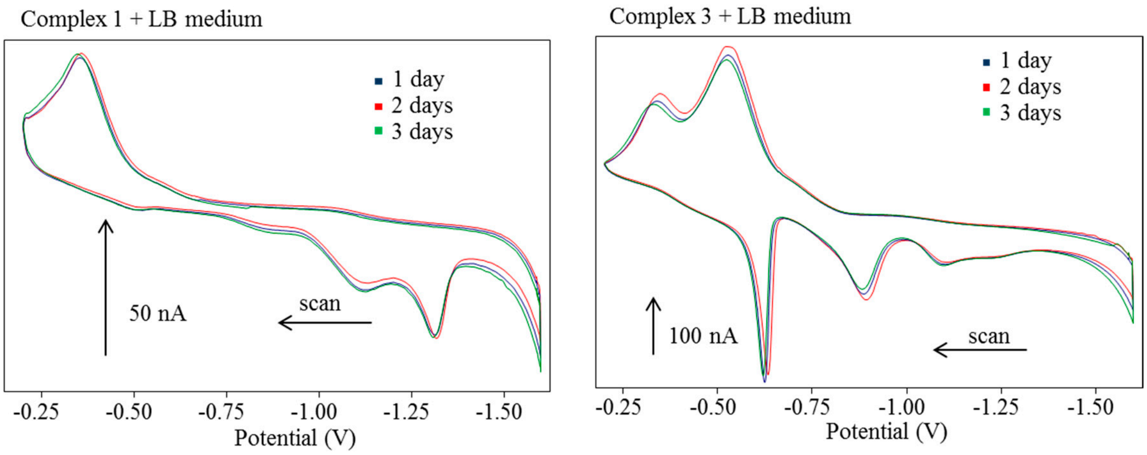

Figure S1. Cyclic voltammograms of the complexes **1** and **3** measured in LB medium.
